# Association of biochemical markers with bone marrow lesion changes on imaging—data from the Foundation for the National Institutes of Health Osteoarthritis Biomarkers Consortium

**DOI:** 10.1186/s13075-023-03253-x

**Published:** 2024-01-18

**Authors:** Shirley P. Yu, Leticia A. Deveza, Virginia B. Kraus, Morten Karsdal, Anne-Christine Bay-Jensen, Jamie E. Collins, Ali Guermazi, Frank W. Roemer, Christoph Ladel, Venkatesha Bhagavath, David J. Hunter

**Affiliations:** 1 Department of Rheumatology, Royal North Shore Hospital, Reserve Road, St Leonards, Sydney, NSW 2065 Australia; 2 Sydney Musculoskeletal Health, The Kolling Institute, School of Medicine, Faculty of Medicine and Health, University of Sydney, Sydney, NSW Australia; 3 Department of Medicine, Duke University School of Medicine, Durham, NC USA; 4 Nordic Bioscience A/S, Herlev, Denmark; 5 Orthopaedic and Arthritis Centre for Outcomes Research, Brigham and Women’s Hospital, Boston, MA USA; 6 Chobanian & Avedisian School of Medicine, Boston University, Boston, MA USA; 7 Department of Radiology, Universitätsklinikum Erlangen & Friedrich-Alexander Universität (FAU) Erlangen-Nürnberg, Erlangen, Germany; 8 CHL4special Consulting, Darmstadt, Germany; 9 Northern Sydney Local Health District, Royal North Shore Hospital, St Leonards, Sydney, NSW Australia

**Keywords:** Osteoarthritis, Biomarkers, Bone marrow lesions

## Abstract

**Background:**

To assess the prognostic value of short-term change in biochemical markers as it relates to bone marrow lesions (BMLs) on MRI in knee osteoarthritis (OA) over 24 months and, furthermore, to assess the relationship between biochemical markers involved with tissue turnover and inflammation and BMLs on MRI.

**Methods:**

Data from the Foundation for the National Institutes of Health OA Biomarkers Consortium within the Osteoarthritis Initiative (*n* = 600) was analyzed. BMLs were measured according to the MRI Osteoarthritis Knee Score (MOAKS) system (0–3), in 15 knee subregions. Serum and urinary biochemical markers assessed were as follows: serum C-terminal crosslinked telopeptide of type I collagen (CTX-I), serum crosslinked N-telopeptide of type I collagen (NTX-I), urinary CTX-Iα and CTX-Iβ, urinary NTX-I, urinary C-terminal cross-linked telopeptide of type II collagen (CTX-II), serum matrix metalloproteinase (MMP)-degraded type I, II, and III collagen (C1M, C2M, C3M), serum high sensitivity propeptide of type IIb collagen (hsPRO-C2), and matrix metalloproteinase-generated neoepitope of C-reactive protein (CRPM). The association between change in biochemical markers over 12 months and BMLs over 24 months was examined using regression models adjusted for covariates. The relationship between C1M, C2M, C3M, hsPRO-C2, and CRPM and BMLs at baseline and over 24 months was examined.

**Results:**

Increases in serum CTX-I and urinary CTX-Iβ over 12 months were associated with increased odds of changes in the number of subregions affected by any BML at 24 months. Increase in hsPRO-C2 was associated with decreased odds of worsening in the number of subregions affected by any BML over 24 months. C1M and C3M were associated with BMLs affected at baseline.

**Conclusions:**

Short-term changes in serum CTX-I, hsPRO-C2, and urinary CTX-Iβ hold the potential to be prognostic of BML progression on MRI. The association of C1M and C3M with baseline BMLs on MRI warrants further investigation.

## Background

Changes in the subchondral bone, including bone marrow lesions (BMLs) on MRI, have been associated with the incidence and progression of knee osteoarthritis (OA) [1]. BMLs are linked with other pathological features in OA [2, 3] and may play a role driving pain symptoms in OA as well as predicting prognosis and treatment outcomes [2]. There are suggestions that a particular phenotype of knee OA patients has involvement of the subchondral bone as part of their disease process [4, 5], and BMLs have been used as a potential treatment response biomarker in trials assessing agents that target subchondral bone resorption including zoledronate [6, 7] and strontium ranelate [8].

As biochemical alterations are thought to precede structural changes related to OA onset [9], there is the assumption that changes in biochemical markers are associated with the development of BMLs [10]. Numerous imaging and biochemical markers have demonstrated prognostic validity for progression in knee OA [11–13]. Further developments in the understanding between biochemical markers and imaging changes will be valuable for identification of biochemical markers that can predict long term imaging changes and thereby impact not only costs and clinical trial efficacy but also serve as a potential intervention focus. If there is a biochemical marker that is predictive of BML presence and progression, it could ultimately become a surrogate marker for BMLs.

The Foundation for the National Institutes of Health (FNIH) OA Biomarkers Consortium conducted a phase I biomarker validation study using a nested case–control sample of symptomatic and/or radiographic knee OA progression within the Osteoarthritis Initiative (OAI) from 2012 to 2015 [14]. The purpose of this project was to establish the prognostic validity of imaging and biochemical markers for knee OA progression. Multiple papers focusing on individual biomarker domains have been published [12, 15–18]. Additional biochemical markers, namely serum matrix metalloproteinase (MMP)-degraded type I, II, and III collagen (C1M, C2M, C3M), serum high sensitivity propeptide of type IIb collagen (hsPRO-C2), and serum matrix metalloproteinase-generated neoepitope of CRP (CRPM), were recently made available to the OA Biomarkers Consortium since these prior publications. These circulating biomarkers are associated with the extracellular matrix and inflammation and have demonstrated associations with different aspects of OA symptomology and pathology [19]. From rheumatoid studies, these biomarkers may reflect bone inflammation [20]; however, their relationship to bone metabolism in OA is unknown.

The intent of this study was to build upon the prior study by Deveza et al. [21] who assessed the association between biochemical markers of bone turnover and bone changes on imaging. At baseline, higher baseline biochemical marker levels of most markers assessed were associated with BMLs, whether it be the maximal size of BMLs or greater number of subregions affected.

The aim of this study was to assess the prognostic value of short-term change (from baseline to 12 months) in biochemical markers as it relates to BML changes on imaging over 24 months. As a secondary aim, we further evaluated the association between biochemical markers of tissue turnover (C1M, C2M, C3M, hsPRO-C2) and inflammation (CRPM) (baseline and time-integrated concentrations (TICs)) and BMLs on imaging at baseline and over 24 months.

## Methods

A supplementary analysis was conducted of baseline, 12 months, and 24 months data from the FNIH Biomarkers Consortium within the OAI.

### Study participants

All 600 participants from the FNIH Biomarkers Consortium OA sample were included in this analysis. This is a case–control design study population, which consists of OA progressors (clinical and radiographic progressors combined), clinical-only progressors, radiographic-only progressors, and non-progressors. The baseline demographic and clinical information was acquired for all participants. The baseline radiographs and MRIs were taken concurrently and reviewed independently by two separate teams of readers. The specifics of the radiograph readings and MRI acquisition have been outlined previously [22]. Eligible participants were those with at least one knee with a baseline Kellgren/Lawrence (KL) grade of 1, 2, or 3. Other eligibility criteria included the availability of radiographs and MRIs, clinical data, and stored biologic specimens. Only participants with the prospective to fulfill criteria for radiological and pain progression (i.e., baseline minimum medial joint space width ≥ 1.0 mm and/or Western Ontario and McMaster Universities Osteoarthritis Index pain scores ≤ 91, 0–100 scale) from baseline to 24 months were selected.

### Semiquantitative MRI analysis of bone marrow lesions

BMLs were scored with the MRI Osteoarthritis Knee Score (MOAKS) instrument [23]. MOAKS utilizes a four-category ordinal scale to score BML size, which encompasses the size of ill-defined and cystic components of BMLs in 15 subregions of the knee: grade 0 = none, grade 1 = less than 33% subregional volume, grade 2 = 33–66% of subregional volume, and 3 = greater than 66% of subregional volume. These 15 subregions are comprised of five subregions in the medial tibiofemoral compartment, five subregions in the lateral tibiofemoral compartment, four subregions in the patellofemoral compartment, and the tibial subspinous subregion. Because only a small percentage of BML at baseline (10.4%) was primarily cystic (MOAKS score 0 and 1) as opposed to ill-defined, this was not considered in the analysis.

The maximum BML size score for the joint was determined as the highest BML grade across the whole knee (ranging from 0 to 3). A total count score ranging from 0 to 15 was calculated for the total number of subregions with presence of BMLs. Consistent with the primary FNIH analysis [16], the number of subregions affected was further categorized into 0, 1, 2, 3, 4, or ≥ 5.

### Assessment of biochemical markers

The biochemical marker samples were collected via morning bloods and second morning void urine at each visit using a standardized protocol. The specimens were stored at − 70° at a commercial specimen repository. Kraus et al. [12] have reported the details and results of the primary main study analyses of the biochemical markers. For the intent of this analysis, the biochemical markers analyzed were a continuation of the markers analyzed in the study by Deveza et al. [21]: serum C-terminal crosslinked telopeptide of type I collagen (CTX-I), serum crosslinked N-telopeptide of type I collagen (NTX-I), urinary NTX-I, urinary CTX-Iα and CTX-Iβ, and urinary C-terminal crosslinked telopeptide of type II collagen (CTX-II). Additional biochemical markers included in the current study are serum type I, II, and III collagen degradation mediated by matrix metalloproteinase (MMP) cleavage (C1M, C2M, C3M), highly sensitive pro-peptide of type IIb collagen (hsPRO-C2), and an MMP-derived degradation fragment of C-reactive protein (CRPM). Precision and accuracy criteria were set at CV < 20% on internal quality control samples as well as on duplicate measures.

For out-of-range low biochemical marker concentration values, values interpolated between zero and the lowest standard were used. With urine samples, the creatinine-adjusted values derived by dividing the urine assay values by the corresponding creatinine level for that sample were used. For hsPRO-C2, the number of samples available at baseline was *n* = 437. For the purpose of this analysis, the missing values were excluded, and only complete data was analyzed.

### Definitions of bone marrow lesion changes over time

**Fig. 1:**
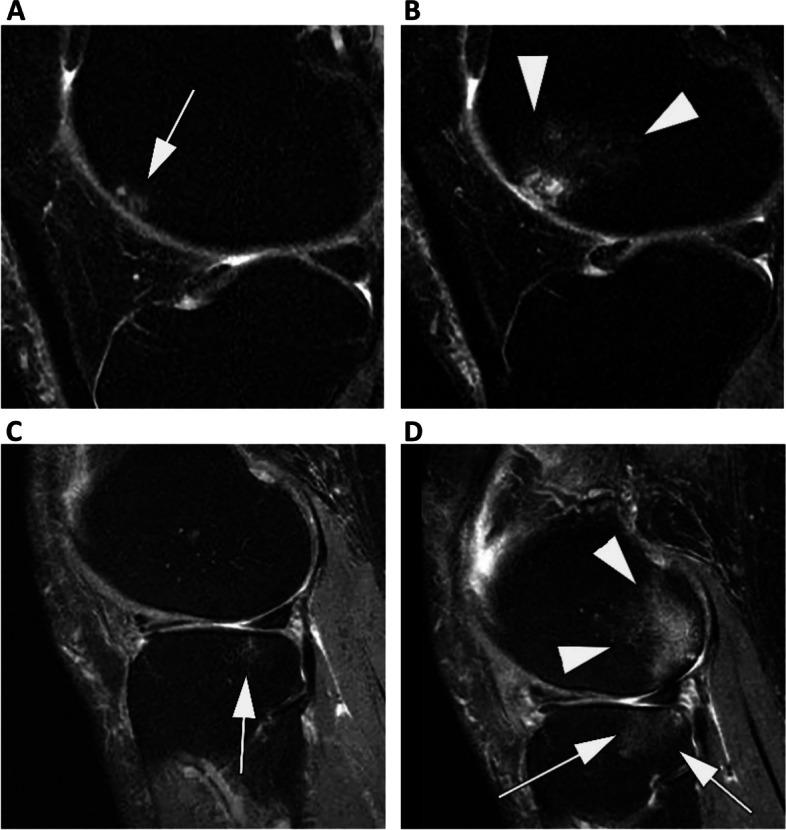
Examples of change in bone marrow lesions (BML). One of the outcome measures was change in maximum size of BML per knee. **A** Baseline sagittal intermediate-weighted fat suppressed MRI shows a small grade 1 BML at the anterior lateral femur (arrow). **B** 24-month follow-up MRI shows increase in size of BML to grade 2 (arrowheads). **C** Another example shows a small grade 1 BML at the posterior lateral tibia at baseline (arrow). **D** 24-month follow-up MRI shows marked increase in tibial BML now comprising the central and posterior tibial subregions (arrows). In addition, there is a new grade 3 BML at the lateral posterior femur (arrowheads) that also involves the central lateral femur (grade 1). This knee shows an increase of number of subregions affected by any BML from 1 subregion at baseline to 4 subregions at 24 months follow-up. Increase in number of subregions affected per knee was the second outcome measure in this study

The maximum worsening in BML size scores was computed across all subregions at baseline and 24 months and was categorized in keeping with a prior study [21] as follows: no change in grade, worsening by 1 grade, or worsening by ≥ 2 grades. Within-grade changes in BML size were not measured in this study. The total number of subregions affected by any BML (i.e., grade > 0) was computed as the difference between the number of subregions affected at 24 months and at baseline. This was categorized as improvement, no change, worsening by 1 subregion, or worsening by ≥ 2 subregions (Fig. 1).

Change scores and time-integrated concentrations (TIC) of biochemical markers over 12 months were utilized to denote their change over 12 months for the prognostic analysis, and TIC over 24 months was used for the concurrent analysis of changes in biochemical markers and BMLs over 24 months.

### Statistical analyses

The outcome for the study was the change in BMLs (maximum size and number of subregions affected); the biochemical markers were used as predictors. The biochemical marker concentrations were standardized to *z* values preceding the analysis for a unit standard deviation (SD) change to be comparable across the biochemical markers. The covariates were age, sex, body mass index (BMI), KL grade at baseline, and participant use of osteoporosis medications (parathyroid hormone in the previous 6 months and bisphosphonate in the previous 12 months) as those might influence bone turnover and may affect bone biochemical marker levels [24–26]. Ordinal logistic regression was used for the ordinal outcome of BMLs, and odds ratio (OR) and 95% confidence intervals (95% CI) for each SD change in biochemical markers were used to assess the strength of associations with BMLs on MRI. The ordinal logistic regression model assumes proportionality of odds. This assumption was tested using the Likelihood ratio test; if the assumption was not satisfied, multinomial regression analysis was used. The analysis was conducted for each individual biochemical marker, and if there are more than one biochemical marker yielding a significant result, they will be included in a multivariable logistic regression model based on a bootstrap cross-validation.

The association between change in biochemical markers (including TICs) over short-term (from baseline to the 12 months follow up visit) and changes in imaging features of BMLs over 24 months (from baseline to the 24-month visit) was assessed. Additionally, for the biochemical markers of C1M, C2M, C3M, hsPRO-C2, and CRPM, cross-sectional analysis of these baseline biochemical marker concentrations and baseline BMLs on MRI, prognostic analysis of their baseline concentrations and changes in BMLs over 24 months, and concurrent analysis of their TICs and changes in BMLs over 24 months were conducted. The TIC values were computed using area under the curve (AUC), measured as per cubic splines rule using the “pkexamine” package in STATA.

Receiver operating characteristics (ROC) curve analysis was conducted to evaluate the diagnostic performance of these biochemical markers to discriminate between knees with BML or without BMLs at baseline. Knees without BMLs were defined as a BML score of 0 in all 15 subregions.

*P* value < 0.05 were considered statistically significant. No adjustments were made for multiple testing. Statistical analyses were conducted using Stata Version 17 (StataCorp. 2021).

## Results

The baseline participant characteristics and biochemical marker concentrations are displayed in Table 1. A higher number of participants were female (58.8%) and in the obese range for BMI. KL grade was predominantly grade 2 or 3 at baseline. Table 2 details the baseline BMLs and their changes over 24 months, with any BMLs seen in 89% of participants at baseline, with grade 3 lesions found in 18.3% of knees. The maximum BML score (size) across all subregions increased by 1 grade in 45.7% and by ≥ 2 grades in 16.6% of knees. Regarding the increase in number of subregions affected by any BMLs, worsening by 1 subregion was observed in 26.8% and by ≥ 2 subregions in 9.8% of knees.

**Table 1:** Study participants characteristics at baseline (*n* = 600)

**Age, mean ± SD years**	61.5 ± 8.9
**Female, %**	58.8
**Body mass index, mean ± SD kg/m**^**2**^	30.7 ± 4.8
**Right knee analyzed, %**	322 (53.7%)
**Kellgren/Lawrence grade, %**	
**1**	75 (12.5%)
**2**	306 (51%)
**3**	219 (36.5%)
**Race, %**	
**African American**	109 (18.2%)
**Asian**	5 (0.8%)
**Others**	11 (1.8%)
**White**	475 (79.2%)
**Medication use, %**	
**Bisphosphonate in last year**	42 (7.8%)
**PTH in last 6 months**	2 (0.3%)
**Biochemical markers, mean ± SD**	
**Serum CTX-I (ng/ml)**	0.39 ± 0.21
**Serum NTX-I (nmole BCE)**	15.11 ± 5.21
**Serum C1M (ng/ml)**	49.87 ± 32.66
**Serum C2M (ng/ml)**	0.48 ± 0.27
**Serum C3M (ng/ml)**	8.26 ± 2.61
**Serum CRPM (ng/ml)**	9.07 ± 8.13
**Serum hsPRO-C2 (ng/ml)**^**a**^	3.72 ± 4.56
**Urinary CTX-II (μg/ml)**	0.30 ± 0.19
**Urinary NTX-I (nmole BCE)**	33.33 ± 17.70
**Urinary CTX-Iα (ng/ml)**	0.43 ± 0.34
**Urinary CTX-Iβ (μg/L)**	2.26 ± 1.76

*PTH* parathyroid hormone, *CTX-I* serum C-terminal crosslinked telopeptide of type I collagen, *NTX-I* serum crosslinked N-telopeptide of type I collagen, *CTX-II* urinary CTX-Iα and CTX-Iβ, urinary NTX-I, urinary C-terminal crosslinked telopeptide of type II collagen, *C1M, C2M, C3M* serum type I, II, III collagen degradation mediated by matrix metalloproteinase (MMP) cleavage, *hsPRO-C2* serum propeptide of type IIb collagen, *CRPM* serum metabolite of C-reactive protein

^a^*n* = 437

**Table 2:** Baseline and changes in bone marrow lesions over 24 months

**Bone marrow lesions, *n* (%)**	**Baseline**		**Change over 24 months**	
**Maximum size**	0	66 (11)	No change	226 (37.7)
	1	223 (37.2)	Worsening by 1 grade	275 (45.7)
	2	202 (33.7)	Worsening by ≥ 2 grades	99 (16.6)
	3	109 (18.3)		
**Number of subregions affected**	0	66 (11)	Improvement	72 (12)
	1	102 (17)	No change	308 (51.3)
	2	128 (21.3)	Worsening by 1 subregion	161 (26.8)
	3	128 (21.3)		
	4	80 (13.3)	Worsening by ≥ 2 subregions	59 (9.8)
	≥ 5	96 (16)		

The association between short-term change in biochemical markers (including TIC over 12 months) and changes in imaging features of BMLs over 24 months are shown in Table 3. Short-term changes in serum CTX-I and urinary CTX-Iβ were associated with increase in the number of subregions affected by any BML at 24 months. For serum CTX-I, a unit SD increase was associated with significantly higher odds of increased number of subregions affected by any BML (OR 1.20 [95% CI 1.02, 1.40]). For a unit SD increase in serum urinary CTX-Iβ, the OR was 1.17 (95% CI 1.00, 1.36). For all biochemical markers, the proportionality of odds assumption was satisfied (*P* > 0.05) apart from serum hsPRO-C2, where further multinominal logistic regression analysis was performed. A unit SD increase in hsPRO-C2 was significantly associated with decreased odds of worsening in the number of subregions affected by any BML by 1 region over 24 months (OR 0.73 [95% CI 0.54, 0.98]) and a non-significant trend for decreased odds of worsening by in two or more subregions (OR 0.18 [95% CI 0.71, 1.96]). There were no significant associations between short-term changes in biochemical markers and 24-month change in maximum BML grade for any biochemical marker. The statistically significant biochemical markers were included in a multivariable logistic regression model analysis, but this did not yield any significant results.

**Table 3:** Association between short-term (0–12 months) change in biochemical markers and long-term (24 months) bone marrow lesion change

	**Serum CTX-I**	**Serum NTX-I**	**Serum C1M**	**Serum C2M**	**Serum C3M**	**Serum CRPM**	**Urine NTX-I**	**Urine CTX-II**	**Urinary CTX-Iα**	**Urinary CTX-Iβ**	^**a**^**Serum hsPRO-C2**
**BMLs**											
Maximum size	1.11 *P* = 0.217 (0.94, 1.30)	1.01 *P* = 0.860 (0.86, 1.19)	1.02 *P* = 0.783 (0.87, 1.21)	1.02 *P* = 0.828 (0.84, 1.24)	1.00 *P* = 0.952 (0.86, 1.17)	0.98 *P* = 0.769 (0.84, 1.14)	0.99 *P* = 0.857 (0.86, 1.14)	1.02 *P* = 0.769 (0.88, 1.19)	0.99 *P* = 0.838 (0.86, 1.13)	1.09 *P* = 0.260 (0.94, 1.28)	0.96 *P* = 0.683 (0.80, 1.16)
	Time-integrated concentrations										
	1.06 *P* = 0.501 (0.90, 1.24)	1.01 *P* = 0.937 (0.86, 1.17)	0.97 *P* = 0.727 (0.83, 1.14)	1.07 *P* = 0.429 (0.91, 1.25)	1.00 *P* = 0.952 (0.86, 1.17)	0.95 *P* = 0.520 (0.83, 1.10)	1.02 *P* = 0.809 (0.88, 1.17)	0.98 *P* = 0.819 (0.84, 1.15)	1.02 *P* = 0.801 (0.89, 1.17)	1.06 *P* = 0.478 (0.90, 1.24)	0.99 *P* = 0.954 (0.84, 1.18)
Number of subregions	1.20 *P* = 0.026 (1.02, 1.40)	1.17 *P* = 0.051 (1.00, 1.37)	1.08 *P* = 0.375 (0.91, 1.28)	0.91 *P* = 0.294 (0.75, 1.09)	1.08 *P* = 0.347 (0.92, 1.26)	0.94 *P* = 0.369 (0.81, 1.08)	0.98 *P* = 0.823 (0.86, 1.13)	1.04 *P* = 0.638 (0.89, 1.21)	0.98 *P* = 0.784 (0.85, 1.13)	1.17 *P* = 0.045 (1.00, 1.36)	^a^Multinomial logistic regression
	Time-integrated concentrations										
	1.06 *P* = 0.487 (0.90, 1.24)	1.05 *P* = 0.551 (0.90, 1.22)	1.10 *P* = 0.261 (0.93, 1.30)	1.13 *P* = 0.142 (0.96, 1.33)	1.06 *P* = 0.482 (0.91, 1.24)	0.97 *P* = 0.712 (0.85, 1.12)	1.02 *P* = 0.784 (0.89, 1.17)	1.03 0.669 (0.86, 1.21)	1.02 *P* = 0.750 (0.89, 1.17)	1.05 *P* = 0.538 (0.90, 1.23)	0.94 *P* = 0.444 (0.79, 1.11)
^a^Multinominal logistic regression											
Improvement No change Worsening 1 subregion Worsening 2 + subregions											1.13 (0.71, 1.79), *P* = 0.602 Ref 0.73 (0.54, 0.98), *P* = 0.039 0.78 (0.71, 1.96), *P* = 0.527

*BMLs* bone marrow lesions, *OR* odds ratio, *95% CI* 95% confidence interval, *CTX-I* serum C-terminal crosslinked telopeptide of type I collagen, *NTX-I* serum crosslinked N-telopeptide of type I collagen, *CTX-II* urinary CTX-Iα and CTX-Iβ, urinary NTX-I, urinary C-terminal crosslinked telopeptide of type II collagen, *C1M, C2M, C3M* serum type I, type II and type III collagen degradation mediated by matrix metalloproteinase (MMP) cleavage, *hsPRO-C2* serum propeptide of type IIb collagen, *CRPM* serum metabolite of C-reactive protein

^a^Multinomial logistic regression analysis conducted as proportionality of odds assumption was not satisfied

When assessing the TIC of the biochemical markers over 12 months and the changes in BMLs over 24 months, there were no significant findings.

For the additional biochemical markers added to the FNIH, the association between these biochemical markers and BMLs on MRI at baseline are shown in Table 4. Serum C3M was associated with significantly higher odds of increased maximum size of BMLs (OR 1.28 [95% CI 1.10, 1.49]). Serum C3M and C1M were associated with significantly higher odds of increased number of subregions affected by any BMLs with an OR of 1.25 (95CI 1.08, 1.44) and 1.16 (95% CI 1.00, 1.34), respectively.

**Table 4:** Association between biochemical markers C1M, C2M, C3M, CRPM, and hsPRO-C2 and BML imaging features at baseline

	^**a**^**Serum C1M**	**Serum C2M**	**Serum C3M**	^**a**^**Serum CRPM**	**Serum hsPRO-C2**
**BMLs**					
Maximum size	^a^Multinomial logistic regression	0.98 *P* = 0.779 (0.84, 1.14)	1.28 *P* = 0.001 (1.10, 1.49)	^a^Multinomial logistic regression	1.01 *P* = 0.943 (0.85, 1.20)
Number of subregions	1.16 *P* = 0.046 (1.00, 1.34)	1.00 *P* = 0.951 (0.85, 1.17)	1.25 *P* = 0.002 (1.08, 1.44)	1.02 *P* = 0.760 (0.89, 1.16)	1.00 *P* = 0.959 (0.84, 1.18)
^a^Multinominal logistic regression					
Maximum size	Ref			Ref	
0 1 2 3	1.14 (0.81, 0.60), *P* = 0.456 1.21 (0.86, 1.70), *P* = 0.274 1.24 (0.86, 1.78), *P* = 0.244			1.25 (0.64, 2.47), *P* = 0.515 1.17 (0.59, 2.35), *P* = 0.641 1.27 (0.64, 2.52), *P* = 0.501	

*BMLs*, bone marrow lesions; *OR*, odds ratio; *95% CI*, 95% confidence interval; *C1M, C2M, C3M*, serum type I, type II and type III collagen degradation mediated by matrix metalloproteinase (MMP) cleavage; *hsPRO-C2*, serum propeptide of type IIb collagen; *CRPM*, serum metabolite of C-reactive protein

^a^Multinomial analysis conducted as proportionality of odds assumption was not satisfied

**Fig. 2:**
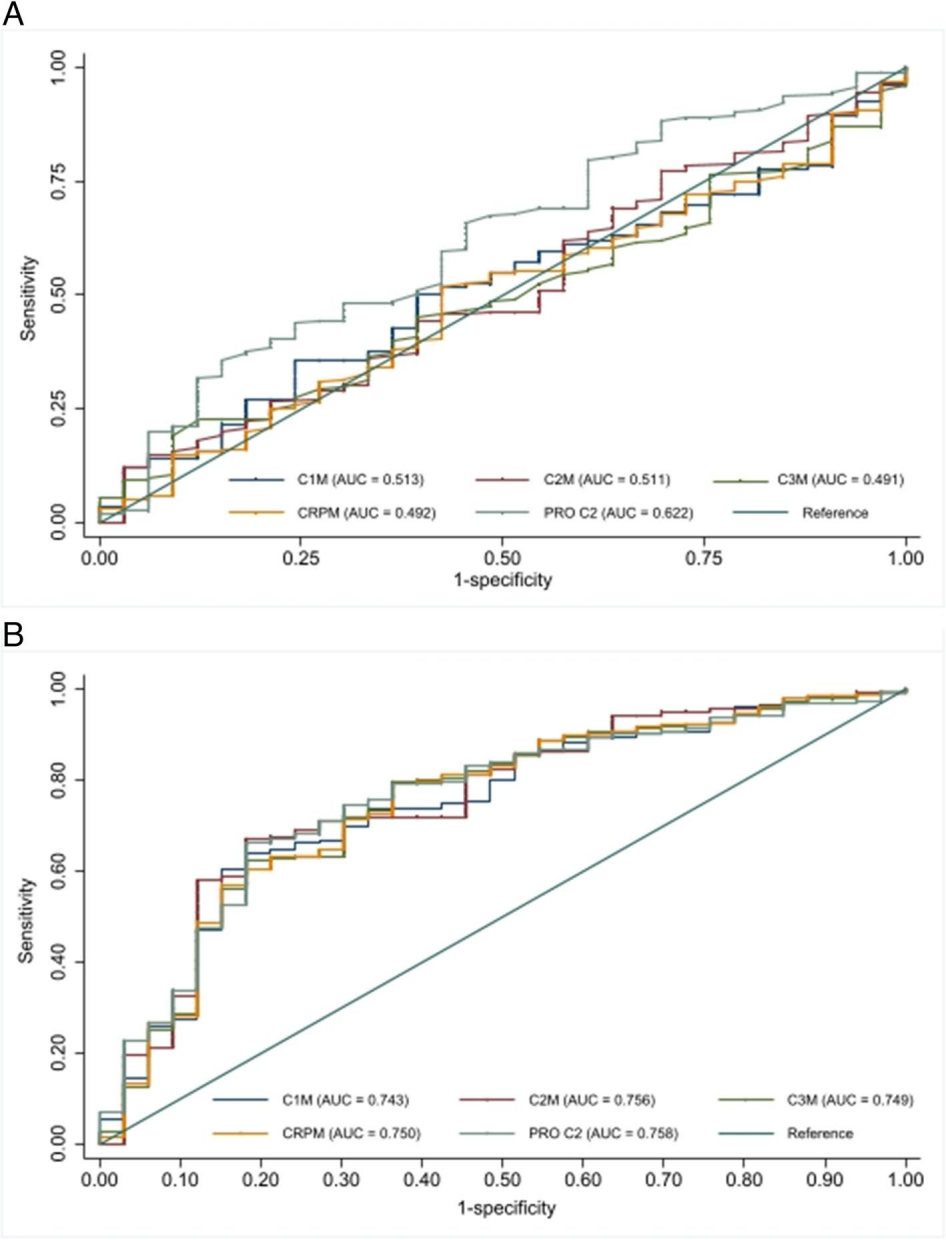
Receiver operating characteristic curves of biochemical markers for predicting the presence and absence of bone marrow lesions at baseline. **A** unadjusted and **B** adjusted for covariates. Serum type I, type II, and type III collagen degradation mediated by matrix metalloproteinase (MMP) cleavage (C1M, C2M, C3M), serum propeptide of type IIb collagen (hsPRO-C2), and serum metabolite of C-reactive protein (CRPM)

The area under the ROC curves (AUCs), utilizing the presence of ≥ 1 subregion with BML versus absence of BML at baseline as the outcome, showed the best diagnostic performance for hsPRO-C2 with an AUC of 0.622 (95% CI 0.50, 0.69). With the addition of covariates, the performance for all the biochemical markers became comparable (Fig. 2).

There were no significant associations seen with baseline biochemical markers of C1M, C2M, C3M, CRPM, and hsPRO-C2 and changes in BML maximum size or number of subregions affected over 24 months (Table 5), and no significant associations were seen with concurrent change analysis of TIC of the biochemical markers (TIC over 24 months) and changes in BML features over 24 months (Table 6). There were weak associations between TIC of serum C2M and hsPRO-C2 and the number of BML subregions affected over 24 months (OR 1.16 [95% CI 0.95, 1.41] and OR 1.13 [95% CI 0.90, 1.43] respectively).

**Table 5:** Association between biochemical markers C1M, C2M, C3M, CRPM, and hsPRO-C2 at baseline and changes in BML imaging features from baseline to 24 months

BMLs	Serum C1M	Serum C2M	Serum C3M	Serum CRPM	Serum hsPRO-C2
Maximum size	0.99	0.95	0.11	0.96	1.02
	*P* = 0.911	*P* = 0.523	*P* = 0.467	*P* = 0.539	*P* = 0.825
	(0.84, 1.17)	(0.79, 1.12)	(0.91, 1.24)	(0.83, 1.10)	(0.85, 1.23)
Number of subregions	1.13	1.12	1.08	0.97	1.06
	*P* = 0.147	*P* = 0.206	*P* = 0.328	*P* = 0.631	*P* = 0.543
	(0.96, 1.34)	(0.34, 1.32)	(0.83, 1.26)	(0.84, 1.11)	(0.88, 1.28)

*BMLs*, bone marrow lesions; *OR*, odds ratio; *95% CI*, 95% confidence interval; *C1M, C2M, C3M*, serum type I, type II and type III collagen degradation mediated by matrix metalloproteinase (MMP) cleavage; *hsPRO-C2*, serum propeptide of type IIb collagen; *CRPM*, serum metabolite of C-reactive protein

**Table 6:** Concurrent change: time integrated concentration of biochemical markers C1M, C2M, C3M, CRPM, and hsPRO-C2 and changes in BML over 24 months

BMLs	Serum C1M	Serum C2M	Serum C3M	Serum CRPM	Serum hsPRO-C2
Maximum size	0.98	0.92	1.08	0.96	0.97
	*P* = 0.858	*P* = 0.411	*P* = 0.345	*P* = 0.528	*P* = 0.799
	(0.82, 1.17)	(0.76, 1.12)	(0.92, 1.27)	(0.83, 1.10)	(0.79, 1.19)
Number of subregions	1.05	1.16	1.05	1.96	1.13
	0.591	*P* = 0.138	*P* = 0.570	*P* = 0.613	*P* = 0.285
	(0.88, 1.25)	(0.95, 1.41)	(0.89, 1.22)	(0.84, 1.11)	(0.90, 1.43)

*BMLs* bone marrow lesions, *OR* odds ratio, *95% CI* 95% confidence interval, *C1M, C2M, C3M* serum type I, type II and type III collagen degradation mediated by matrix metalloproteinase (MMP) cleavage, *hsPRO-C2* serum propeptide of type IIb collagen, CRPM serum metabolite of C-reactive protein

## Discussion

In this study, there were several biochemical markers whose short-term change predicted the BML progression in terms of the number of subregions affected on imaging at 24 months—namely, the type I collagen markers indicative of bone resorption: serum CTX-I and urinary CTX-Iβ. The association of short-term change in these biochemical markers and longer-term BML changes can be extrapolated to the prior findings from the FNIH OA Consortium, wherein these biochemical makers were associated with disease progression by Kraus et al. [12], who studied radiographic joint space loss progression and persistent pain as outcome measures. However, in this study, other type I collagen markers, including serum and urinary NTX-I, urinary CTX-α, and type II collagen degradation marker urinary CTX-II, did not demonstrate associations with BML progression. The discordance potentially relates to the assessment of different outcomes, radiographic joint space narrowing (JSN), and pain in the FNIH study versus a specific bone-related outcome, BML, in this study. In addition, the imaging outcome timepoints differed for the studies; progression from baseline to 24–48 months was the outcome in FNIH as opposed to baseline to 24 months in this study. Even though the FNIH study did not assess progression of BMLs directly, it can be assumed that radiographic structural progression in part will be linked to BMLs on MRI. Several longitudinal studies have found BML severity to be positively related with cartilage defect and volume loss, joints space narrowing, and joint replacement [3, 27, 28].

Despite CTX-II being one of the best performing prognostic biomarkers for disease progression in relation to KL grade, JSN, or predicted total joint replacement [12, 29, 30], this study did not detect any association between short-term changes in urinary CTX-II with BML changes over 24 months. Again, this may relate to outcome timepoints. Although traditionally considered a biochemical marker of cartilage degeneration, baseline urinary CTX-II has been detected in association with baseline BMLs [21, 31, 32]. As BMLs have been demonstrated to have areas of high metabolic activity; these lesions show reduced bone marrow volume with replacement by dense fibrous connective tissue, increased vascularization, hyaline cartilage, and fibrocartilage [2, 33]. Therefore, CTX-II potentially has a role in bone metabolism. However, its association with longitudinal BMLs changes to date has not been detected [21, 34]. This is in accord with the current study.

With the additional biochemical markers available to the FNIH, short-term changes in serum hsPRO-C2, a biochemical marker related to type II collagen (type IIB propeptide fragment) synthesis, were associated with decreased odds of worsening in the number of BML subregions affected at 24 months. Serum PRO-C2 was developed and proposed for the estimation of cartilage formation [35]. Its levels are significantly higher in the control cohort compared to the OA cohort in the oral calcitonin trials; it is inversely associated with 2-year radiographic progression of joint space narrowing [36, 37]. Higher PIIANP (a type II collagen synthesis marker) similarly was inversely associated with OA progression in the FNIH cohort [12]. These type II collagen biomarkers may be objective indicators of low cartilage repair endotype [35] in need of an anabolic stimulus given that those with low levels of PRO-C2 (compared to those with levels above the median) appeared to lose more cartilage thickness over time and grow more cartilage in response to sprifermin versus placebo [38]. To date, there are no data regarding the association of PRO-C2 and BMLs. BMLs are associated with cartilage damage in the same subregion and predict cartilage loss longitudinally [39]. Given the reduced odds of the number of BML subregions affected at 24 months with short-term biochemical marker changes, higher levels of PRO-C2 may be indicative of an OA cohort that is less likely to progress. Conversely, those with low levels are the at-risk group in need of intervention targeting cartilage formation and/or maintenance.

Apart from serum CTX-I and PRO-C2, and urinary CTX-Iβ, no other short-term changes in biochemical markers were found to be prognostic of BML changes at 24 months. Potential reasonings behind the negative findings relate to the biochemical markers being systematically measured in the bloods and that BMLs are localized lesions. The release of biochemical markers into the system may not be cleared into the blood and may be dependent on synovial vascularity [40]. Even though many of the biochemical markers analyzed had prognostic associations with cartilage degradation and bone turnover, it is unclear whether these changes relate to the OA process or are due to cross-reactivity of the epitope [41]. A concern with biochemical markers is that they may be more dynamic in nature and may be influenced by activity or injury [12, 42]. The relatively weak or lack of association in this study may indicate the heterogenous nature of OA, and the biochemical marker changes may only partially explain the changes captured on MRI overtime [42].

As part of the secondary aim, the evaluation of baseline association between biochemical markers of tissue turnover and inflammation and BMLs on imaging at baseline and over 24 months demonstrated association of baseline biochemical markers and baseline BMLs. C1M and C3M were significantly associated with presence of BMLs at baseline with C3M being associated with both maximum size and a greater number of subregions involved. C1M is a biochemical marker for extracellular matrix turnover of type I collagen, a main constituent of bone and connective tissue. As bone consists of mainly type I collagen, which is resorbed predominantly by cathepsin K, in rheumatoid arthritis (RA) and OA, there is a shift towards an MMP-driven degradation process. C1M detection in arthritic conditions is thought to relate to MMP mediated type I destruction at the matrix and at the synovial level [43]. In RA studies, C1M predicts progression of joint destruction and response to treatment and is considered to be connected with the ongoing process of joint deterioration [20, 44, 45]. In OA studies, C1M is associated with pain outcomes [46] and has shown pharmacodynamic responses to anti-inflammatory (anti-interleukin-1 alpha/beta variable domain immunoglobulin) and diet/exercise interventions [47, 48]. Association between C1M and erosive hand OA has been observed [49], indicating that C1M can be a potential driver of inflammation related bone and soft tissue turnover in an OA subtype. In this study assessing BMLs, the association with number of BML subregions may reflect local inflammation. Overall, the relationship between C1M with OA progression remains unclear with no association with C1M observed in terms of incidence or progression of OA in the Rotterdam Study [50]. No association was found in this study in the prognostic analyses.

C3M is a type III collagen neoepitope generated by MMP. Previous data have shown that C3M was associated with disease activity and current disease state in RA in relation to synovial inflammation [20, 51]. Elevated concentrations of C3M were found in those with OA when compared to healthy controls [52], but no association with pain have been found with change in this marker over time [48]. Correlation of C3M with radiological features have been conflicting: one study showed correlation of C3M with JSN [53], a second study showed correlation with osteophyte as well as serum and synovial fluid CD163 (a marker of pro-inflammatory macrophages) [54], while a third study showed no correlation with knee radiological severity [55]. Vertebral endplate bone marrow lesions (Modic changes) have been associated with C3M [56]. This suggests that C3M in OA may reflect not only synovitis but also inflammatory niches in bone related to BMLs. Type III collagen is one of the key bone marrow matrix constituents, and the bone marrow fibrosis seen relative to bone marrow lesion formation may be exhibited by the changes in C3M levels.

Together with C1M and C3M, CRPM, an MMP-dependent degradation of CRP in serum, appears to also reflect tissue inflammation in individuals with knee and/or hip pain likely related to primary OA [57]. Elevated CRPM has been observed in OA patients with higher C1M and CRP levels, supporting the concept of inflammation in a subset of the OA population [52]. No association with CRPM has been found in this study despite high levels being found to be prognostic of incident knee OA [50, 58]. As C1M and C3M but not CRPM were found to have associations with BMLs, these biochemical markers may reflect distinct inflammatory domains or niches in OA [57].

Similarly, with C2M, an MMP-mediated inter-helical degradation of Col2, this biochemical marker has shown a positive association with KL grade structural changes [52]. As it is predominantly a biochemical marker of cartilage turnover, it is consistent with this study where no associations with BMLs are found both cross-sectionally, and longitudinally.

While we found the short-term changes of biochemical markers at 12 months provided some significant associations with BML changes at 24 months, these findings should be considered preliminary as no associations were found when the TIC of these biochemical markers were assessed at 12 months. However, these metabolites may behave more like disease activity markers; thus, use of TIC may be less valid.

There are some limitations to this study. Firstly, the analyses were conducted as a post hoc analysis on a subsample of the FNIH cohort, which consists of OA progressors. The individual groups were not taken into consideration in this study, and it is unclear whether the study population will reflect the general knee OA population. Additionally, the study only assessed knee status and its association with biochemical markers. OA involvement at other joint sites, depending on their disease status, may potentially influence the levels of the systemic measurements of these biochemical markers. It has been shown that different joints, i.e., hip, knees, hands, contribute to urinary CTX-II levels [59]. Furthermore, biochemical markers such as CTX-I may also originate from non-articular tissue, reflecting general bone turnover. It would have been ideal to have bone mineral density data, given the influence it can have on biochemical markers, especially those of bone turnover, to use as an adjustment covariate in this study. Lastly, as no adjustments were made for multiple testing, the occurrence of type I error is unable to be excluded.

In conclusion, improved understanding of BMLs could ultimately be another step towards identification of subjects at risk of symptomatic and structural OA progression. If biochemical marker measurements can predict BML progression on MRI, they have the potential to improve personalized care, allow for the identification of new treatment targets, and pave the way for clinical trial efficiency. This study highlights that short-term changes in biochemical markers could potentially provide prognostic information regarding progressive BML changes on MRI. Several statistically significant associations with baseline biochemical markers and BMLs are found, suggesting connective tissue turnover involvement in the pathogenesis of BMLs. These results require further validation and verification as well as application to progressive changes in other imaging findings in OA.

## Data Availability

All data are available from the OAI of the FNIH (https://data-archive.nimh.nih.gov/oai).

## Abbreviations

AUC: Area under the curve
BMLs: Bone marrow lesions
C1M, C2M, C3M: Matrix metalloproteinase degraded type I, II, and III collagen
CI: Confidence interval
CRPM: Matrix metalloproteinase-generated neoepitope of C-reactive protein
CTX-I, CTX-II: C-terminal crosslinked telopeptide of type I and type II collagen
FNIH: Foundation for the National Institutes of Health
hsPRO-C2: High sensitivity propeptide of type IIb collagen
JSN: Joint space narrowing
KL: Kellgren/Lawrence
MMP: Matrix metalloproteinase
MOAKS: MRI Osteoarthritis Knee Score
OR: Odds ratio
OA: Osteoarthritis
ROC: Receiver operating characteristics
SD: Standard deviation
TIC: Time-integrated concentration
